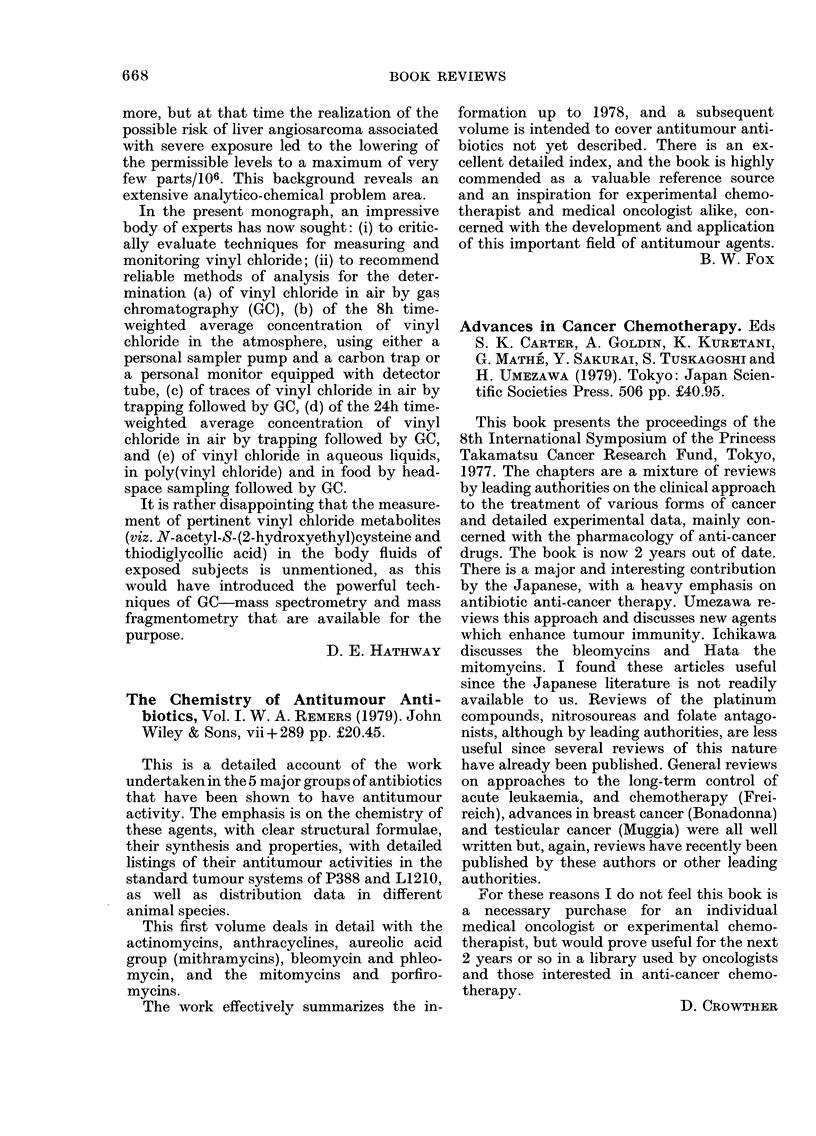# The Chemistry of Antitumour Antibiotics, Vol. I

**Published:** 1979-10

**Authors:** B. W. Fox


**The Chemistry of Antitumour Antibiotics,** Vol. I. W. A. Remers (1979). John Wiley & Sons, vii+289 pp. £20.45.

This is a detailed account of the work undertaken in the 5 major groups of antibiotics that have been shown to have antitumour activity. The emphasis is on the chemistry of these agents, with clear structural formulae, their synthesis and properties, with detailed listings of their antitumour activities in the standard tumour systems of P388 and L1210, as well as distribution data in different animal species.

This first volume deals in detail with the actinomycins, anthracyclines, aureolic acid group (mithramycins), bleomycin and phleomycin, and the mitomycins and porfiromycins.

The work effectively summarizes the information up to 1978, and a subsequent volume is intended to cover antitumour antibiotics not yet described. There is an excellent detailed index, and the book is highly commended as a valuable reference source and an inspiration for experimental chemotherapist and medical oncologist alike, concerned with the development and application of this important field of antitumour agents.

B. W. Fox